# Supplementary material for: Addressing the Real-World Challenges of Immunoresistance to Botulinum Neurotoxin A in Aesthetic Practice: Insights and Recommendations from a Panel Discussion in Hong Kong
Source: Toxins (Basel). 2023 Jul 12;15(7):456. doi: 10.3390/toxins15070456 (PMC10467074; doi:10.3390/toxins15070456)

File S1. Methods for the consumer studies

The 2018 and 2021 consumer studies were online surveys with country/territory representative samples, utilising similar methodology and respondent screening criteria, conducted by a consumer research agency. Data were collected using a semi-structured quantitative questionnaire. The list of countries/territories where the study was conducted and the sample breakdown are listed in the table below.

Table S1. Respondent sample size by countries in the consumer study

		2018 survey	2021 survey
Total		2201	2441
	Australia	500	505
	Hong Kong	250	255
	Korea	520	625
	Taiwan	370	375
	Thailand	360	365
	Singapore	100	105
	Philippines	-	105
	Indonesia	-	106
	India	101	-

Participant inclusion criteria were:

- Aged between 21 to 55 years
- Must have personally been administered with 3 or more botulinum toxin treatments
- Must not be a Xeomin (incobotulinumtoxinA, INCO) user
- Must fall into the following monthly household income categories
    - Australia: >AUD5,000
    - Hong Kong: >HKD30,000
    - Korea: >KRW4,000,000
    - Taiwan: >TWD70,000
    - Thailand: >THB50,000
    - Singapore: >SGD7,000
    - Philippines: >PHP50,000
    - Indonesia: >IDR5,000,000
    - India: >IDR200,000

Table S2. Respondent characteristics in the 2018 and 2020 consumer studies.

**Characteristics**	**2018** (N=2201)	**2021** (N=2441)
Gender		
Male	15%	15%
Female	85%	85%
Age, average (years)	37 years	37 years
21–25	4%	7%
26–30	20%	19%
31–35	23%	21%
36–40	19%	20%
41–45	15%	17%
46–50	14%	10%
51–55	6%	7%
Average monthly household income		
Australia	AUD 13,774	AUD 15,443
Hong Kong	HKD 67,080	HKD 76,569
India	INR 379,951	-
Indonesia	-	IDR 28,113,208
Korea	KRW 8,156,731	KRW 10,540,800
Philippines	-	PHP 87,810
Singapore	SGD 17,005	SGD 20,715
Taiwan	TWD 117,622	TWD 124,227
Thailand	THB 92,263	THB 89,027

Figure S1. Emotional impact of diminishing BoNT-A efficacy (overall)

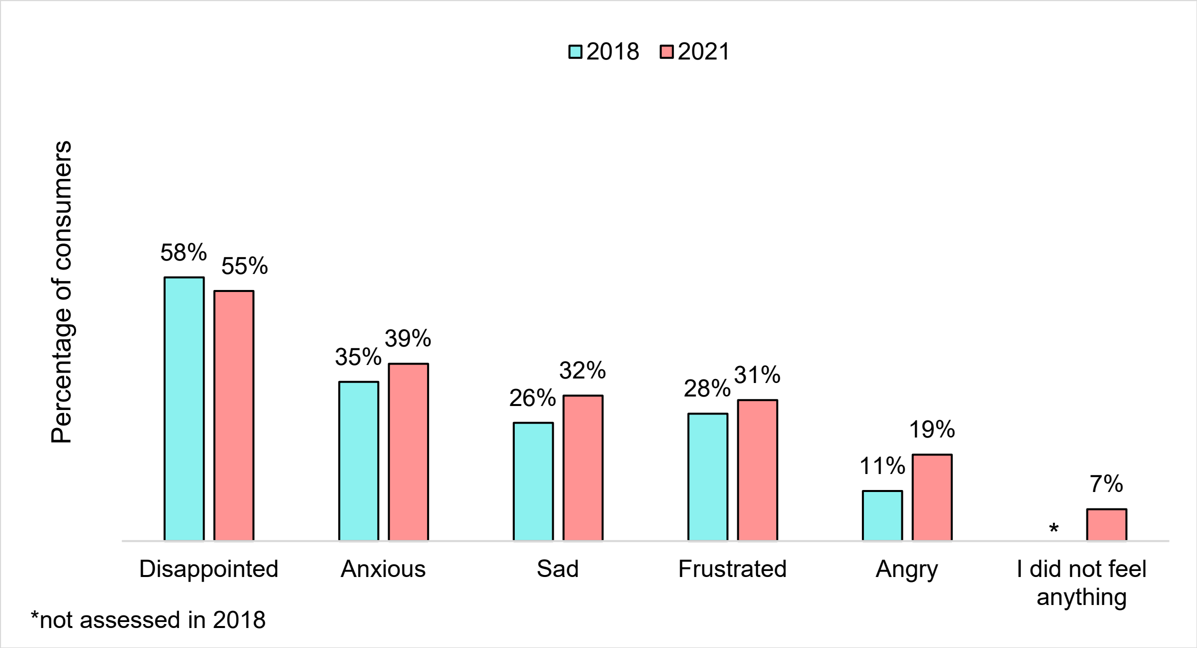

Figure S2. Consumer awareness and experience of diminishing BoNT-A treatment efficacy (Hong Kong data). (A) Awareness of diminishing efficacy and associated signs/symptoms. (B) Experienced diminishing efficacy

(A)

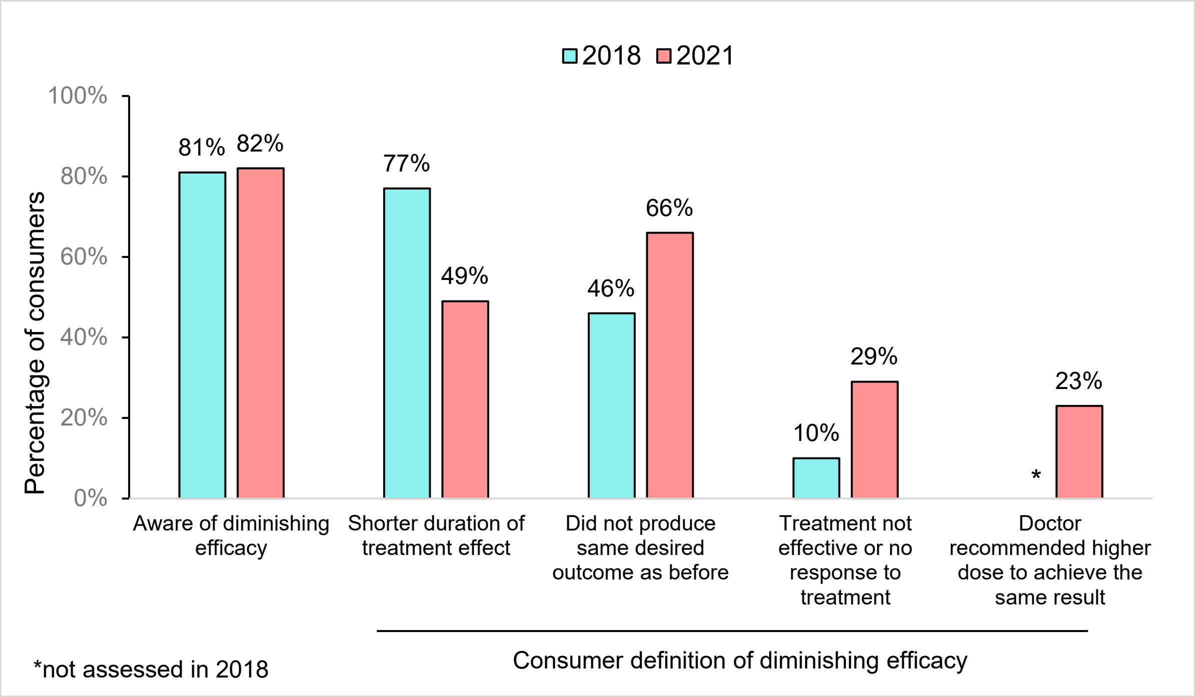

(B)

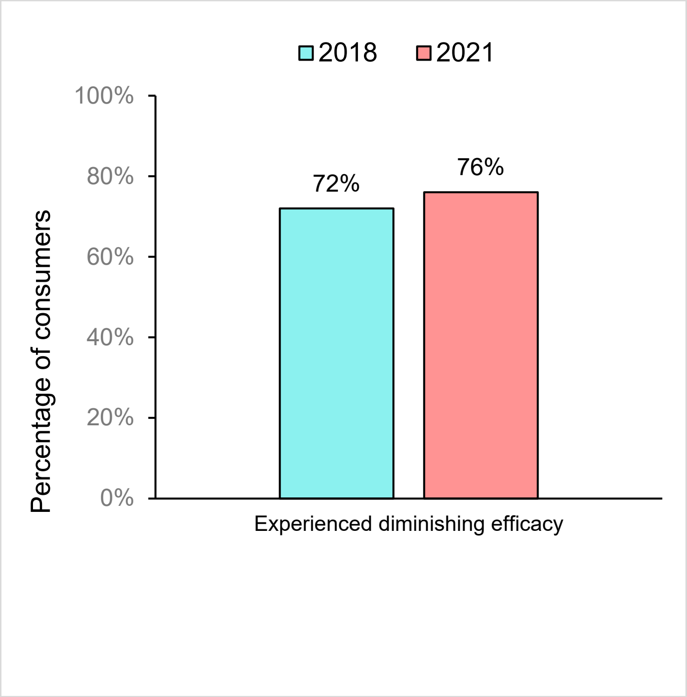

Figure S3. Emotional impact of diminishing BoNT-A treatment efficacy (Hong Kong data)

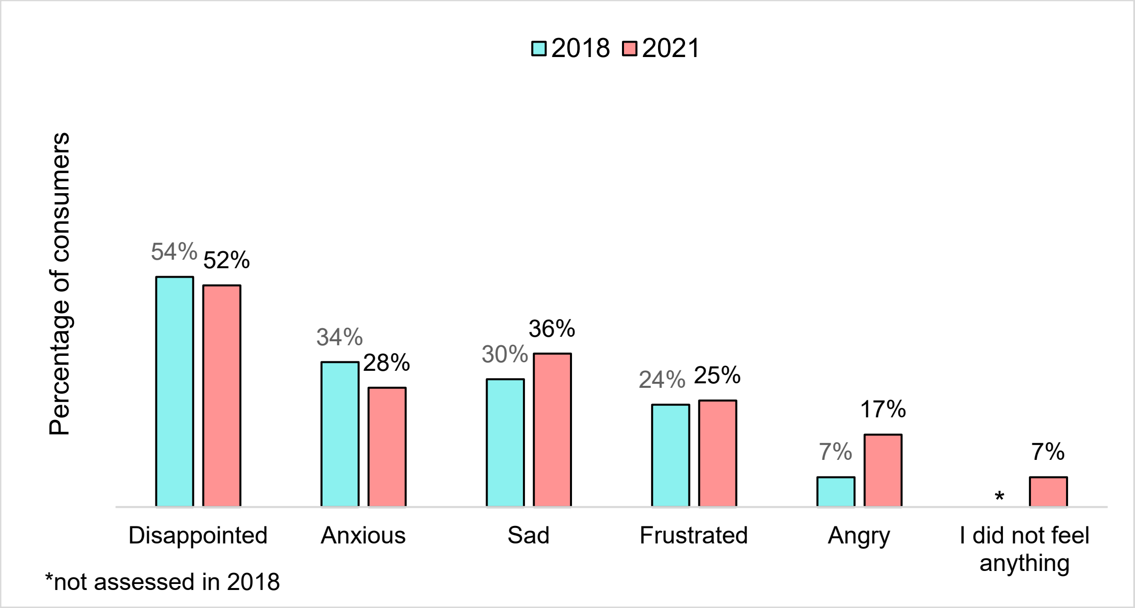

Supplement: Supplementary file 1 [file toxins-15-00456-s001.zip › toxins-2456074-supplementary.pdf]
